# Eight Elements in Soils from a Typical Light Industrial City, China: Spatial Distribution, Ecological Assessment, and the Source Apportionment

**DOI:** 10.3390/ijerph16142591

**Published:** 2019-07-20

**Authors:** Yujie Pan, Hongxia Peng, Shuyun Xie, Min Zeng, Changsheng Huang

**Affiliations:** 1 Research Center of Spatial Planning and Human-Environment System Simulation, China University of Geosciences, Wuhan 430074, China; 2 Department of Geography, School of Geography and Information Engineering, China University of Geosciences, Wuhan 430074, China; 3 School of Earth Sciences, China University of Geosciences, Wuhan 430074, China; 4 Wuhan Geological Survey Center of China Geological Survey, Wuhan 430205, China

**Keywords:** geostatistical analysis, assessment, sources, Zhongshan City, Guangdong–Hong Kong–Macao Greater Bay Area

## Abstract

Contamination with the eight elements, Hg, As, Cr, Cu, Ni, Pb, Zn, and Cd, is a serious concern in Zhongshan, which is a typical light industrial city, China. 60 surface soil samples were collected to investigate the concentrations, spatial distribution, human health risk, and sources of these elements in the soils in Zhongshan. The concentrations of the eight elements were analyzed while using ordinary kriging analysis, pollution load index (PLI), potential ecological risk index (RI), human health risk, correlation analysis, and factor analysis. The mean concentrations of the tested elements, excluding Pb and As, were higher than the soil background values in the Pearl River Delta. The spatial distribution of the tested elements revealed a zonal distribution pattern and high values in several areas. The mean PLI and RI indicated slight and moderate risk levels. Health risk assessment demonstrates that both children and adults were more exposed to Cu than to Cr, As, and Cd. However, the associated carcinogenic risk is acceptable. Hg that originated from human activities; As, Cr, Cu, Ni, and Cd originated from industrial activities; and, Pb and Zn originated from transportation activities. Cd was the main pollutant in the study area and it was present at higher concentrations when compared with those of the other elements. Therefore, Zhongshan should encourage enterprises to conduct industrial transformation to control the ecological risk.

## 1. Introduction

With global socioeconomic development, elemental pollution has gradually become a key factor that threatens the soil ecological environment and endangers human health [1,2]. Therefore, the pollution of these elements in the soil is a source of global concern [3,4]. Elemental pollutants accumulate in the soil because of natural and human activities [5]. In addition, Elemental pollutants can easily enter the human body through ingestion, dermal contact, or breathing, and they can threaten health [6,7]. Low concentrations of these elements can cause various health issues, such as renal dysfunction, endocrine disorders, reproductive dysfunctions, and cancers [8,9]. Therefore, the study of the spatial distribution, ecological risks, and sources of these elements in soil is required [10,11].

Numerous studies have demonstrated that these elements are increasingly introduced into the environment by industrial processes, the application of agrochemicals, waste disposal, and wastewater irrigation [12,13,14,15]. Therefore, it is crucial to investigate the sources of these elements to ensure that further pollution can be prevented and reduced. Multivariate statistics and geostatistical analysis are paradigmatic methods for studying the sources and spatial differentiation of elements in soils and they have been extensively used to determine the sources of these elements in soil [16,17,18,19]. Ordinary kriging (OK) has been extensively used to study the spatial structure and variability of materials in soil since the advent of soil science research in the 1980s [20,21]. Previous research has shown that it is more suitable to assess the health risk that is presented by these elements in soils to human based on human health risk assessment [22,23,24]. The method has been extensively applied to study these elements pollution in different regions.

After the reform and opening up of China, the state strongly supported the economic development of the Pearl River Delta region, especially after the national strategy of Guangdong–Hong Kong–Macao Greater Bay Area was proposed. The Guangdong–Hong Kong–Macao Greater Bay Area is one of the most important bay areas in China and it is the main route to ensure the participation of south China in the global economy. It is one of the four largest bay areas in the world, being comparable with the bay areas of New York, San Francisco, and Tokyo. Zhongshan City is located in the geometric center of this area, playing an important role in “connecting east to west” and gradually developing into a typical light industrial city in China. Numerous light industries manufacturing products, such as textiles, home appliances, food, and household chemicals, have majorly contributed to the economic development of Zhongshan City; however, they have extensively impacted the ecological environment of the region. Limited studies are available regarding pollution in Zhongshan City. Therefore, comprehensive domestic studies regarding this are required. This study was performed to address the research gaps and to better understand pollution in the northern part of the Zhongshan City. We performed ordinary kriging analysis, pollution load index (PLI), potential ecological risk index (RI), and human health risk assessments to provide a comprehensive reference for local governments. This study area has not been previously studied. This study aims to provide worthwhile information regarding the spatial distribution and pollution levels and any possible sources of eight elements. The results of the present study can provide reference information to facilitate land planning and management activities in regions with light industries that are contaminated with these elements.

## 2. Materials and Methods

### 2.1. Study Area

**Figure 1:**
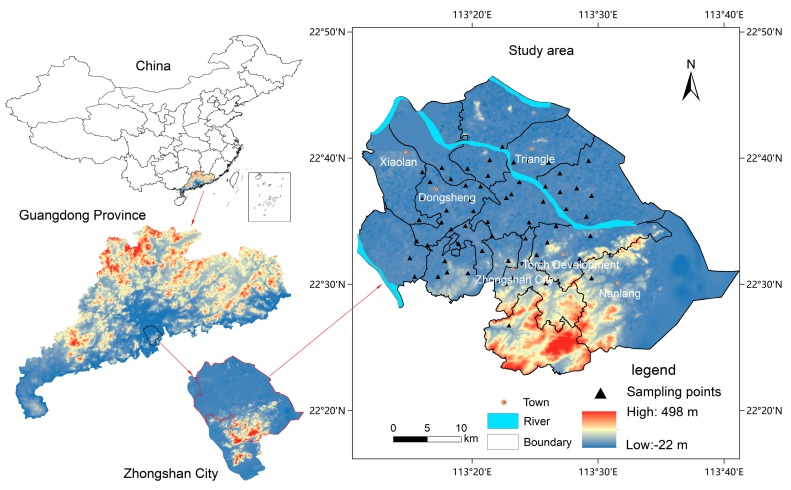
Location of Zhongshan City and soil sample collection sites.

The study area was located in northern Zhongshan at the mouth of the Pearl River, South China (113°9′2″E–113°46′E, 22°11′12″N–22°46′35″N; Figure 1). The study area includes Nanlang Town, Zhongshan City, Torch Development Zone, Triangle Town, Dongsheng Town, Xiaolan Town and other Towns of Zhongshan City. The area experiences a subtropical monsoon climate, along with an annual mean temperature of approximately 21 °C. The study area belongs to the marine-continental interfacial sedimentary landform in the front margin of the Pearl River Delta plain. The terrain is low, flat, open, and slightly inclined toward the southeast. The stratigraphic division of the area belongs to the Dongjiang section of the South China strata area, and the Proterozoic, Cretaceous, and Quaternary strata were exposed on the surface.

### 2.2. Sample Collection and Analysis

The surface soil (0–20 cm) samples were collected from the study area while using the grid and random sampling methods. The study area was a rectangle measuring 4 × 15 km^2^ and the sampling sites were randomly selected and arranged in 60 grids of 1 km^2^. During the field sampling stages, the sampling sites were selected based on the topography while using maps and the global positioning system. Furthermore, the grid coordinates of each sampling point were recorded and the sampling site and surrounding environment were described. The road, village, and factory locations were recorded in detail to indicate the characteristics of the sampled area. Before sampling, we removed the upper 2 cm of the surface soil and collected the fresh soil samples. The samples weighed >1000 g and they were naturally dried, passed through a 20 mesh (<0.84 mm) sieve, and transferred to the laboratory for processing through a 200 mesh (<0.074 mm) sieve for analysis. Soil pH was determined while using 10 mesh (<2 mm) sieved samples. The requirements of antipollution measures were ensured during sample collection, transportation, processing, and assembly, such as each sample being sealed and a hygrometer was installed in each sample box. Figure 1 illustrates the soil sample collection sites.

The test indicators included soil physical and chemical indicators (soil moisture content, soil pH, cation exchange capacity, and soil organic matter content) and soil inorganic indicators (Hg, As, Cr, Cu, Ni, Pb, Zn, and Cd). The background control values were conducted by using Chinese Local Standards (Risk Screening Values for Soil Heavy Metal, The Pearl River Delta Area (DB44/T 1415/2014)) [25]. Hg, As, Cr, Cu, Ni, Pb, Zn, and Cd are 0.13, 25.00, 77.00, 32.00, 28.00, 60.00, 97.00, and 0.11, respectively. The Guangzhou Mineral Resources Supervision and Inspection Center of the Ministry of Land and Resources conducted the present element analysis. Hg and As were tested while using atomic fluorescence spectrometry (AFS-933, Jitian Corporation, Shanghai, China) [12], whereas Cr, Cu, Ni, Pb, Zn, and Cd were tested while using inductively coupled plasma mass spectrometry (ICP-MS, Agilent 7700X, Agilent Technologies, Santa Clara, CA, USA) [12].

### 2.3. Methods

#### 2.3.1. Statistical Analysis

Factor analyses were used to identify the variables that explained most of the variance in the data, and the mean, minimum, maximum, median, standard deviation, and coefficient of variation were calculated. Correlation analyses were used to investigate the associations between elements, and multivariate analyses were used to identify the major sources of these elements in the study area [26].

#### 2.3.2. Ordinary Kriging

Ordinary kriging (OK) analysis assumes that changes in data have either normal or log-normal distributions and the expected values of regionalized variables are not known [27]. The interpolation process of OK is similar to that of the weighted moving average, and the weights are derived from the kriging equations. The Equation for this model is as follows:

(1) $$Z^{*}{(x}_{0})\;=\;\overset{n}{\underset{i=1}{\sum }}{\;\varphi }_{i}{Z(x}_{i})$$

where $Z^{*}{(x}_{0})$ is the linear prediction value and ${Z(x}_{i})$ is the observed value. n is the total number of measured samples, i is the samples identifier, and $\varphi_{i}$ is the optimal weight value that aids in an unbiased prediction with minimum variance.

#### 2.3.3. Pollution Load Index

Tomlinson first described the PLI, which is a comprehensive measure of the pollution by more than one element [28]; it is expressed, as follows:

(2) $$\mathrm{PI}\;=\;\frac{C_{n}}{\mathrm{GB}};\;\mathrm{PLI}\;=\;\sqrt[{n}]{{\mathrm{PI}}_{1}{\;\times \;\mathrm{PI}}_{2}\;\times \;\ldots {\;\times \;\mathrm{PI}}_{n}}$$

where PI is the single pollution index, GB values of the geochemical background (mg/kg), and $C_{n}$ is the content of heavy metal in soil (mg/kg). Additionally, n is the number of analyzed elements. In the present study, the background value of the Pearl River Delta region was used as the evaluation standard; PLI is the pollution load index of a sampling site and n is the number of evaluated elements.

#### 2.3.4. Potential Ecological Risk Index

RI is a method that was employed by the Swedish scientist Hakanson to evaluate the elemental pollution and ecological hazards on the basis of the principles of sedimentology [29]. He combined ecological effects, environmental effects, and element toxicity to consider the general migration and transformation trends of these elements in soils and sediments, in addition to the sensitivity of the areas that were assessed for heavy metal pollution. The RI of an element is divided into comprehensive indicators that reflect the potential effect of these elements on the ecological environment. RI is expressed as follows:

(3) $$\mathrm{PI}\;=\;\frac{C_{n}}{\mathrm{GB}}{;\;E}_{r\;}^{i}{=\;T}_{r}^{i}\;\times \;\mathrm{PI};\;\mathrm{RI}\;=\;\overset{n}{\underset{i\;=\;1}{\sum }}{\;E}_{r}^{i}$$

where PI is the single pollution index, GB values of the geochemical background (mg/kg), and $C_{n}$ is the content of heavy metal in soil (mg/kg) [30]. $E_{r}^{i}$ is the single RI. $T_{r}^{i}$ is the toxicity response coefficient of element i. The toxicity response coefficient is based on Hakanson’s element toxicity response coefficient standards, which can be given as follows: Hg = 40, As = 10, Cr = 2, Cu = 5, Ni = 5, Pb = 5, Zn = 1, and Cd = 30. RI is the comprehensive RI of soil. Table 1 summarizes the description of ecological assessment level.

#### 2.3.5. Human Health Risk Assessment

Health risk assessment can be done based on the non-carcinogenic and carcinogenic risk for both adults and children. Hazard identification, exposure assessment, dose–response assessment, and risk characterization are the key elements of health risk assessment [31]. Chronic daily intake (CDI) (mg/kg/day) through incidental ingestion (CDI_ingest_) and dermal contact (CDI_dermal_) was determined by the following Equations to predict the human health risk that is caused by exposure to these elements. Table 2 shows all the definitions and reference values for these elements.

(4) $${\;\mathrm{CDI}}_{\mathrm{ingest}}\;=\;\frac{\mathrm{CS}\;\times \;\mathrm{IR}\;\times \;\mathrm{CF}\;\times \;\mathrm{FI}\;\times \;\mathrm{EF}\;\times \;\mathrm{ED}}{\mathrm{BW}\;\times \;\mathrm{AT}}$$

(5) $${\mathrm{CDI}}_{\mathrm{dermal}}\;=\;\frac{\mathrm{CS}\;\times \;\mathrm{CF}\;\times \;\mathrm{SA}\;\times \;\mathrm{AF}\;\times \;\mathrm{ABS}\;\times \;\mathrm{EF}\;\times \;\mathrm{ED}}{\mathrm{BW}\;\times \;\mathrm{AT}}\;$$

(1) Non-Carcinogenic Risk

Hazard quotient (HQ) is the non-carcinogenic risk, where a value of one refers to the threshold reference value that was suggested by the US Environmental Protection Agency (USEPA). If HQ is <1, no potential non-carcinogenic risks occur in the study area. *RfD* is the reference dose of metal and Table 3 presents the values. Hazard index (HI) is characterized by the sum of HQ, which indicates the cumulative non-carcinogenic risk. HQ and HI are expressed, as follows:

(6) $${\mathrm{HQ}}_{\mathrm{ingest}}{\;=\;\mathrm{CDI}}_{\mathrm{ingest}}{/\mathrm{RfD}}_{\mathrm{ingest}}$$

(7) $${\mathrm{HQ}}_{\mathrm{dermal}}{\;=\;\mathrm{CDI}}_{\mathrm{dermal}}{/\mathrm{RfD}}_{\mathrm{dermal}}$$

(8) $$\mathrm{HI}\;=\sum {\mathrm{HQ}\;=\;\mathrm{HQ}}_{\mathrm{ingest}}{+\;\mathrm{HQ}}_{\mathrm{dermal}}$$

(2) Carcinogenic Risk

Carcinogenic risk (CR) is estimated from the product of the CDI and the slope factor (SF) over a lifetime. The SF plays a key role in the convention that the daily toxin intake results in an incremental risk of an individual developing cancer and Table 3 presents the values. The sum of CR characterizes the total cancer risk (TCR).

(9) $${\mathrm{CR}}_{\mathrm{ingest}}{\;=\;\mathrm{CDI}}_{\mathrm{ingest}}{\;\times \;\mathrm{SF}}_{\mathrm{ingest}}$$

(10) $${\mathrm{CR}}_{\mathrm{dermal}}{\;=\;\mathrm{CDI}}_{\mathrm{dermal}}{\;\times \;\mathrm{SF}}_{\mathrm{dermal}}$$

(11) $$\mathrm{TCR}\;=\sum \mathrm{CR}=CR_{\mathrm{ingest}}{\;+\;\mathrm{CR}}_{\mathrm{dermal}}$$

## 3. Results and Discussion

### 3.1. Descriptive Statistical Analysis

Table 4 presents the descriptive statistics of the concentrations for eight elements in 60 soil samples. The arithmetic means and medians for these elements in surface soils that were obtained from the study area were low, and the influences of extreme values were minimal. The coefficient of variation indicates the discrete characteristics of the statistical data and it is a key parameter for measuring the distribution of an element in the soil. The coefficients of variation of these elements in soil in northern Zhongshan City were ranked as follows: Hg > Pb > Cd > Cu > Zn > Cr > Ni > As. In this study, the coefficients of variation for all the tested elements, excluding Hg, were below 100, which indicated the low dispersion of these element concentrations in the surface soil. Generally, study areas have distribution signatures for these element combinations, which result from variations in anthropogenic and natural sources.

In recent years, studies of element pollution levels have been performed in other cities in the Greater Bay Area of Guangdong, Hong Kong, and Macau. Thus, the element concentrations in Zhongshan were compared with those in these cities (Table 5) [30,31,32,33,34,35,36]. The mean values of eight investigated elements, excluding As and Pb, were higher than the soil background values in the Pearl River Delta, which indicated that these elements accumulated with economic development. Except for Jiangmen and Zhaoqing, the concentrations of these elements in other cities of the Greater Bay Area is similar to the trend of heavy metal content in the study area, which may be related to the rapid urbanization and exponential industrial agglomeration in these areas after the reform and opening up.

### 3.2. Spatial Distribution

**Figure 2:**
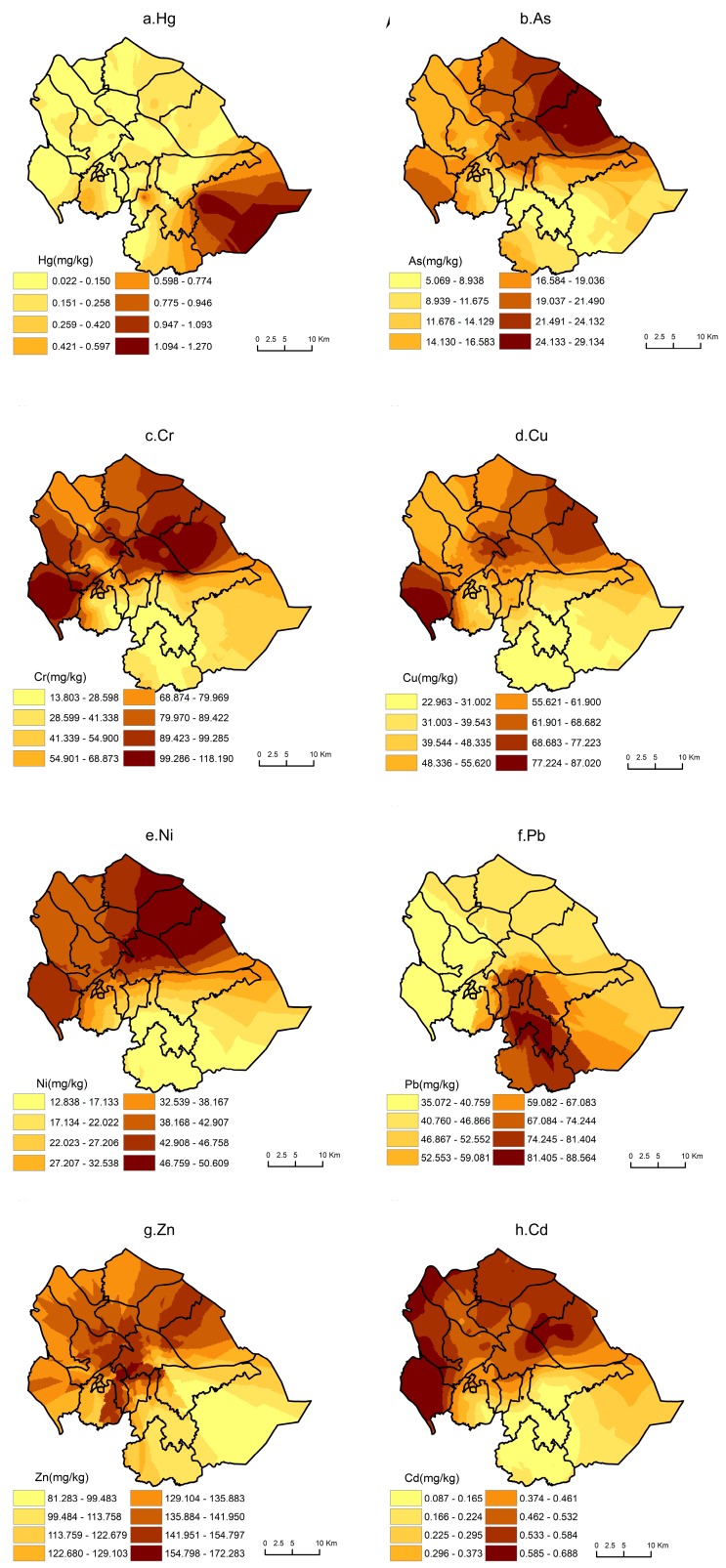
Spatial distributions of elements concentrations in the study area. The maps were generated using ordinary kriging.

The geostatistical method has been extensively used to study the distribution of the pollution in soil. The present study analyzed the spatial distributions of the concentrations of eight elements in soils based on Equation (1), and ArcGIS 10.2 (http://www.esri.com/software/arcgis, ESRI, Redlands, California, CA, USA) was used to construct a spatial distribution map and to interpolate the concentrations of the common elements in the study area using OK. The result is shown in Figure 2. Overall, the spatial distribution patterns of these elements in soil were zonal and showed high concentrations in some areas, which indicated that human activities in the area had negative effects on concentration in soils. The Hg concentration gradually decreased from the southeast hilly region to the northern plain region (Figure 2a). As, Cu, and Ni had generally similar spatial distribution patterns in the study area (Figure 2b,d,e), with the high-value area mainly distributed in the popular town, port town, and Shaxi Town and the low-value area mainly distributed in the hills in southeastern Zhongshan. High Cr concentrations were distributed in populated towns and low concentrations were found in the hilly areas to the southeast of the Zhongshan City (Figure 2c). The high-value area of Pb in shallow soil was distributed in the main urban area of Zhongshan, whereas the low-value area was mainly distributed in the northern plain (Figure 2f). The high-value area of Zn in shallow soil was distributed in eastern Zhongshan, whereas the low-value area was distributed in the Zhongshan Torch Development Zone (Figure 2g). The high-value area of Cd in shallow soil was distributed in the northern plain area, where the town population was the highest; however, the low-value area was distributed in the hilly area in southeastern Zhongshan, with the lowest concentrations being observed in Shantou Village (Figure 2h).

The spatial distributions of these elements suggested that the slightly polluted areas were primarily distributed in the populated towns, port towns, and in the northern plains. However, the soils in the southern hilly area are relatively clean and were less affected by human activities. Intensive human activity may be a reasonable cause of heavy metal pollution. Previous studies have shown that unreasonable human activities, such as random discharge of sewage and waste residue, are important causes of the pollution.

### 3.3. Ecological Risk Assessment

#### 3.3.1. Pollution Load Index Assessment

**Figure 3:**
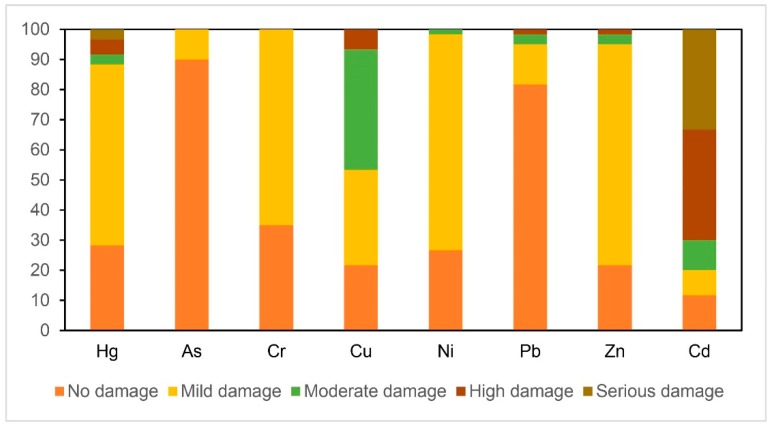
The ratio of the number of different pollution levels of single pollution index to the total number of samples (%).

On the basis of Equation (2) of PLI, the PI of each element in the study area was calculated. The mean PI values from the highest to the lowest were Cd (3.96), Cu (1.77), Hg (1.44), Ni (1.37), Zn (1.35), Cr (1.00), Pb (0.84), and As (0.70). The ratios of different pollution levels of each element are obtained, according to Table 1. Figure 3 shows the result. This ratio equals the number of samples of different contamination levels for each element divided by total number of samples of 60. The results showed that most As and Pb in the samples were nonpolluting or slightly polluting. Among all the samples, the proportions of samples in which Hg was nonpolluting and mildly, moderately, severely, and very strongly polluting were 28.33%, 60.00%, 3.33%, 5.00%, and 3.34%, respectively, Cr, Cu, Ni, and Zn were mild pollutants, and Cd was a severe and strong pollutant in 36.67% and 33.33% of samples, respectively.

**Figure 4:**
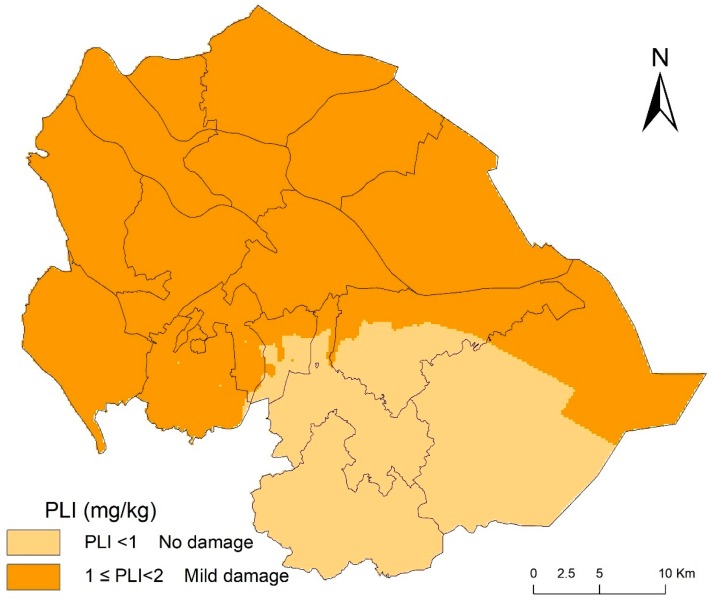
Spatial distributions of pollution load indexes for eight elements in Zhongshan.

The PLI ranges in the present study were from 0.23 to 1.82, with a mean of 1.25, which indicated mild pollution. The number of PLI samples that were associated with nonpolluting and slightly polluting elements were 25% and 75% of all samples, respectively. The OK classification of PLI in the study area and the PLI spatial distribution map were obtained based on the results of PLI and index classification. Figure 4 shows the result. The total PLI values of the present elements in Zhongshan indicated a high potential risk due to human activities.

When comparing with the soil ecological risks of other cities in the Greater Bay Area, it can be found that there are certain differences in the ecological risks of heavy metals. For example, the ecological risk of Dongguan is mainly concentrated on Hg, while Zhongshan is mainly concentrated on Cd [32]. However, overall, the ecological risks are low in all cities in the Greater Bay Area.

#### 3.3.2. Potential Ecological Risk Index Assessment

The single potential ecological risk index (ERI) and RI of eight elements in northern Zhongshan were calculated based on equation (3), and an ecological risk assessment was performed according to Hakanson potential ecological risk grading standards. The average ERI values for the eight elements in the study area were as follows: Cd (118.84), Hg (57.47), Cu (8.88), As (7.02), Ni (6.84), Pb (4.22), Cr (2.01), and Zn (1.35). The potential ecological risks for all As, Cr, Cu, Ni, Pb, and Zn samples were either risk-free or had low-risk levels. The ERI of Cd was the highest among the eight elements based on the ratio of the number of different pollution levels in ERI samples of various elements to the total number of samples, which indicated that the pollution from Cd was more serious than pollution from the remaining elements in all of the samples. In addition, the extent of ecological damage associated with all samples was extreme. The ERI of Hg was second only to Cd, and moderate and severe pollution levels accounted for 71.67% of all the samples. The ERI of the other six elements was low, mainly posing a slight risk.

**Figure 5:**
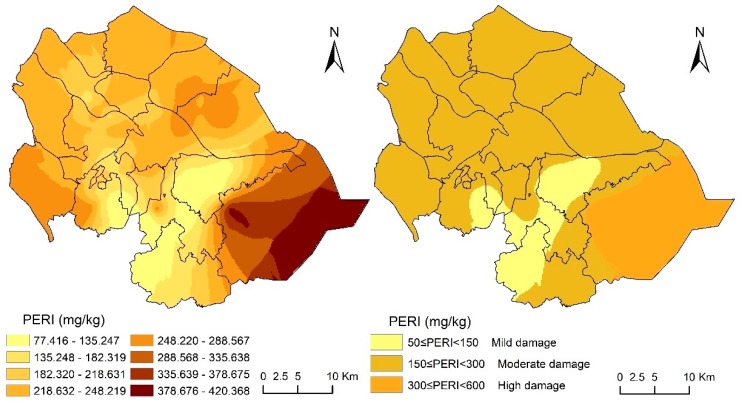
Spatial distributions of the comprehensive potential ecological risk index for eight elements in Zhongshan.

The mean RI was 206.64, which represents a moderate ecological risk, with a range of 29.61–462.67. The samples with mild and moderate pollution accounted for 90% of all samples, according to the RI assessment criteria. The slightly polluted areas were mainly distributed in the transitional areas from the popular towns, port towns, and hilly areas in the northern plains to the plains, which were associated with severe risk levels. Figure 5 shows the result. The RI of the soil in the northern part of Zhongshan showed a slight and moderate spatial distribution risk profile. Therefore, the government should implement several control measures and strict rules to reduce emissions.

#### 3.3.3. Human Health Risk Assessment Children and Adults

**Figure 6:**
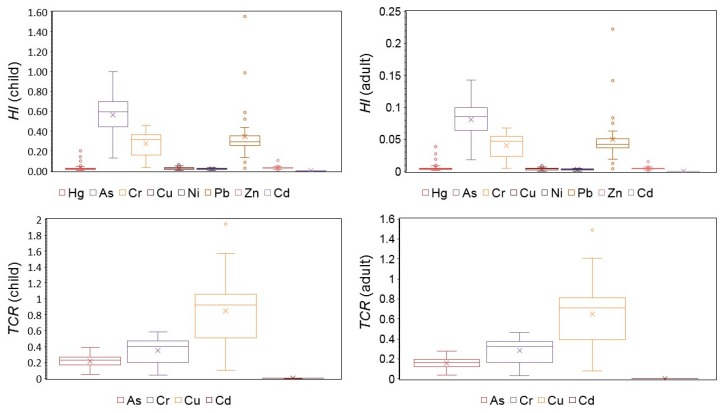
The hazard index (HI) and total cancer risk (TCR) value for human health risk with respect to both children and adults.

Figure 6 presents the HI and TCR values for human health risk for both children and adults. The HI values ranged from 0.000,080,13 to 1.552,602,74 for children and from 0.000,011,46 to 0.222,499,02 for adults, respectively. The HI values for children and adults were found in the descending order of As > Cr > Zn > Hg > Pb > Cu > Cd. The results suggested that the HI values for children of element As and Cr were higher than 1.0, which indicated that there was a potential non-carcinogenic risk for children, and the HI values for adults were lower than 1.0, showing that there was no non-carcinogenic risk for adults. These results suggest that children face greater health risks than adults.

The TCR ranges of As, Cr, Cu, and Cd on children were (0.0497–0.3861) × 10^−4^, (0.0413–0.5849) × 10^−4^, (0.1046–1.9435) × 10^−4^, and (0.0001–0.0038) × 10^−4^. For adults, the TCR values of As, Cr, Cu, and Cd were (0.0358–0.2778) × 10^−4^, (0.0329–0.4659) × 10^−4^, (0.0803–1.4922) × 10^−4^, and (0.0001–0.0031) × 10^−4^. The TCR values for adults were smaller than those for children. The USEPA set the maximum acceptable risk level of 1 × 10^−4^. TCR less than 1 × 10^−6^ have no dramatic effects on human health, whereas TCR exceeding 1 × 10^−4^ can be considered to be unacceptable [31]. The average TCR values for children and adults suggested that the CR that was caused by Cu is unacceptable, whereas that for Cr is acceptable; As and Cd may pose no CR. These findings demonstrate that both children and adults were more easily exposed to Cu than Cr, As, and Cd. However, overall, the risk is acceptable.

The results of human health risk in the Zhongshan City showed that not only was the CR in the region that as similar to those for other cities in the Greater Bay Area, but that it also exhibited some differences [39]. As shown in a study of Dongguan, As and Cr could potentially lead to heightened CRs following oral ingestion and inhalation [32]. In addition, the result of non-carcinogenic risk values indicated that children were facing a slight threat from As and Cr. From the study of Shenzhen, the total non-carcinogenic hazard index was 0.648 for the non-cancer risk assessment results [34]. The performances of five elements is Pb > As > Ni > Cu > Cd. The degree of harm in all three exposure routes was characterized by ingestion > skin contact > respiratory inhalation. The total risk of carcinogenesis was 4.79 × 10^−15^ for carcinogenic risk assessment. The CR of three carcinogenic elements was As > Ni > Cd. These results suggest no connection between these elements and cancer risks in the industrial area.

Overall, Cd in the study area is the major cause of contamination risk. Industrial activity is most likely to affect the risk factor. These results are in accordance with the findings of Chen et al., who report that Cd and Hg are major potentially toxic elements in Chinese soil [40]. The speedy development of China’s economy and exponential agglomeration and development of the industry have greatly increased the discharge of industrial and urban wastewater, waste gas, and waste residue, since the reform and opening up.

### 3.4. Source Apportionment

#### 3.4.1. Correlation Analysis

A correlation analysis was used to identify the sources of the eight elements (Table 6). As, Cr, Cu, Ni, Zn, and Cd were found to be significantly correlated, with the Cr–Ni and Ni–Cd correlation coefficients being 0.937 and 0.800 at a significance level of 0.01, which indicated that they were highly correlated and they may have had a similar source. The lesser correlation between Hg and the other seven elements suggested that they originated from diverse sources in the soils that were studied. Several previous reports have confirmed that these elements primarily originate from anthropogenic activities [31,32]. Consequently, it could be further inferred that there is some relation between the sources of most of these elements in this study and human activities.

#### 3.4.2. Factor Analysis

SPSS 17.0 (SPSS Inc., Chicago, IL, USA) was used to analyze the concentrations of eight elements in the surface soil samples from in northern Zhongshan in the present study. The polluting sources of these elements in the soil were identified. The Kaiser–Meyer–Olkin (KMO) [41] and Bartlett’s spherical tests were also performed for element concentrations [42] of 60 samples. The KMO value was 0.754, the correlation probability of the Bartlett spherical test was zero, which was less than the significant horizontalization of 0.05, and the element concentrations in the 60 samples were considered to be appropriate for factor analyses; Table 7 presents the results.

Factor analysis applies eigenvalues >1, and three principal factors are analyzed under the premise of 85.749% of cumulative variance. The total cumulative variance contribution rate before and after the rotation did not change, which indicated that the maximum orthogonal rotation of the variance resulted in no loss of information. Following rotation, the variance contribution rate of principal factor 1 was 53.686%, the variance contribution rate of principal factor 2 was 19.314%, and the variance contribution rate of principal factor 3 was 12.749%. Therefore, the three factors were deemed to be the major sources of pollution.

When comparing Table 8 before and after rotation, the variable result of the factor load matrix exhibited a slight change before and after factor rotation, which indicated a correlation between the original variable and the common factor. The larger the factor load value, the stronger the correlation between the common factor and original variable. Therefore, the orthogonal factor solution indicated that factor F1 was a combination of As, Cr, Cu, Ni, and Cd; factor F2 was a combination of Pb and Zn; and, Hg represented factor F3.

#### 3.4.3. Source Identification

As, Cr, Cu, Ni, and Cd were the major loads in factor 1, and this observation is consistent with the results of correlation analyses. Cr and Ni remain the major components of stainless-steel manufacturing and they are mainly used for processing. Cu mainly originates from sewage that is discharged from the electronics manufacturing companies and metallurgical industries. Cd was the most polluting element in the study area and it was mainly distributed near the Triangle Town. Cd pollution is mainly from electroplating and coating and wastewater discharge from mine smelting. The high-value areas of As, Cr, Cu, Ni, and Cd were consistent and they were mainly distributed in the northern plains. The distributions of the pollution-control enterprises in northern Zhongshan are consistent with this result. Therefore, factor 1 could be an industrial factor. Industrial pollution is mainly concentrated in the vicinity of the Xiaolan and Jiwa Waterways on the northern plains. Industrial discharges of “three wastes” (Exhaust gas, waste water, waste residue) and element deposits in rivers lead to pollution in the soil. Further, in factor 2, Pb and Zn had higher factor loading, and the high anomaly areas were mainly distributed in Zhongshan. Exhaust emissions from motor vehicles are the major cause of Pb pollution, and Zn-containing dust from tire wear is the major source of Zn. Therefore, factor 2 may primarily relate to transportation. The wear of tires produces large amounts of dust in the urban area of the Zhongshan City. Vehicles also emit large quantities of exhaust gas, which is an important non-point pollution source and it is responsible for the entry of large amounts of the eight elements into soils. These results emphasize the need for increased soil protection and pollution control in the Zhongshan City. Hg had a high factor load with respect to factor 3, and the high anomaly area was identified as the Tuxi Economic Cooperative of Guantang Village in Nantang Town and southern Zhongshan, which was a polluted area. Consumption and construction wastes in the area had direct impacts on the Hg concentrations in the soil. Therefore, factor 3 can be considered to be an artificial factor. In addition, large quantities of domestic garbage, construction waste, and garbage incineration were discovered in the study area during the investigation.

The light industry has been a pillar of the Zhongshan City and it has been a major driving force for its economic development. The content characteristics, distributions, ecological risks, and sources of the eight present elements are very different to those that are related to the heavy industries in cities. For example, the mean concentrations of Hg, As, Cr, Cu, Ni, Pb, Zn, and Cd in Tangshan were 0.06, 5.89, 36.98, 22.42, 16.81, 22.93, 70.31, and 0.15 mg/kg, respectively [17]. Zn had the highest concentration among them. This is primarily related to the coal production and utilization and agricultural activity of this city. Based on different pollution indices, the result of research in Xiangtan highlighted that Cd and Zn were the major contributors to the ecological risk, and the cancer risk of Cd indicated an unacceptable degree in this area [5]. This may be related to the discharge of the untreated industrial wastewater. The severe contamination of nearby farmland may be through water migration, and through the food chain into the grain and the human system. From these comparisons, we conclude that the pollution from soil elements is related with the industrial activities, sewage irrigation, and applications of agrochemicals. However, the element pollution sources in light industrial cities, such as Zhongshan, are mainly based on the human, industrial, and transportation activities.

In recent years, the national strategy of the Guangdong–Hong Kong–Macao Greater Bay Area has been put forward, such as building a modern industrial system with international competitiveness and developing a high-quality living circle strategy that is suitable for living, working, and traveling. Zhongshan’s traditional industries, including those that provide home appliances, lighting, hardware, and food, are undergoing upgrade and transformation. Simultaneously, the three strategic emerging industries, including high-end equipment manufacturing, new-generation information technology, and health care, are developing and growing. This strengthens the industrial connection with other parts of the Greater Bay Area and it also improves its economic strength. The resulting economic growth has gradually led to improved environmental awareness among residents.

It is noteworthy that the limited data may not completely represent the overall soil pollution scenario in Zhongshan. Few relevant studies in other cities in the Guangdong–Hong Kong–Macao Greater Bay Area are needed; the data in this study do not sufficiently represent the situation. In the future studies, more appropriate methods will be developed to address the limitations that are imposed by scarce data, and quantitative risk analyses will be performed [43,44]. Additionally, we can try to analyze new sources of pollution, such as road dust. However, this limitation is not big enough to have an impact on the general assessment results as the research methods that were employed in the selected studies are widely accepted by the scientific community.

## 4. Conclusions

The concentrations, spatial distributions, ecological risks, and sources of eight elements were investigated in this study. Despite the limitations of this study, it provides the first systematic description of pollution levels and the associated health risks of eight elements in the soils of the Zhongshan City, which is a typical light industrial Chinese city. Based on our pollution and health risk assessments, we conclude that the soils surrounding the study area are seriously polluted with metal and metalloid elements that are emitted from human, industrial, and transportation activities. Furthermore, soil pollution from these elements will pose carcinogenic and non-carcinogenic health risks in the future, especially in children and those that live in the most severely polluted regions. This study provides quantitative evidence that tight regulation of effluent emissions in light industrial cities is required to protect the residents from the elemental pollutants in China’s environment.

## Figures and Tables

**Table 1:** Evaluation criteria for different evaluation methods.

Assessment Methods	No Damage	Mild Damage	Moderate Damage	High Damage	Serious Damage	Extreme Damage
CF	CF < 1	1 ≤ CF < 2	2 ≤ CF < 3	3 ≤ CF < 5	CF ≥ 5	-
PLI	PLI < 1	1 ≤ PLI < 2	2 ≤ PLI < 3	PLI ≥ 3	-	-
RI	ERI < 10 RI < 50	ERI < 40 RI < 150	40 ≤ ERI < 80 150 ≤ RI < 300	80 ≤ ERI < 160 300 ≤ RI < 600	160 ≤ ERI < 320 600 ≤ RI < 1200	ERI ≥ 320 RI ≥ 1200

CF, contamination factor; PLI, pollution load index; RI, comprehensive potential ecological risk index; ERI, single potential ecological risk index.

**Table 2:** Definition and reference value of some parameters for health risk assessment.

Parameter	Definition	Value
CS	The element concentration in the sediment (mg/kg)	Measured Value
IR	The ingestion rate (mg/day)	Child: 200 mg/day Adult: 100 mg/day
CF	The conversion factor (kg/mg)	1 × 10^−6^ kg/mg
FI	Fraction ingested from the contaminated source (unitless)	1.0
EF	The EF of the CDI_ingest_ is the exposure frequency (days/year)	350 days/year
ED	ED is the exposure duration (years)	Child: 6 years Adult: 30 years
BW	The average body weight (kg)	Child: 20 kg Adult: 70 kg
AT	The average time (days)	Non-carcinogenic: ED × 365 days/years Carcinogenic: 70 years × 365 days/years
SA	The exposed surface area of skin (cm^2^/event)	Child: 2800 cm^2^ Adult: 5700 cm^2^
AF	AF is the skin adherence factor (mg/cm^2^)	Child: 0.2 Adult: 0.07
ABS	The dermal absorption factor (unitless)	0.001
EF	EF of CDI_dermal_ is the exposure frequency (events/year)	1 events/day × 350 days/year

**Table 3:** Slope factor (SF) for carcinogenic elements and RfD for non-carcinogenic elements.

Elements	SF (mg/kg·d)^−1^		RfD mg/(kg·d)	
	SF_ingest_	SF_dermal_	RfD_ingest_	RfD_dermal_
Hg	-	-	3.00 × 10^−4^	2.13 × 10^−5^
As	1.50 × 10^0^	3.66 × 10^0^	3.00 × 10^−4^	3.00 × 10^−4^
Cr	0.50 × 10^0^	20.00 × 10^0^	3.00 × 10^−3^	7.5 × 10^−5^
Cu	1.70 × 10^0^	4.25 × 10^1^	2.00 × 10^−2^	5.40 × 10^−3^
Ni	-	-	2.00 × 10^−2^	8.00 × 10^−4^
Pb	-	-	1.40 × 10^−3^	5.25 × 10^−4^
Zn	-	-	4.00 × 10^−2^	1.20 × 10^−2^
Cd	5.01 × 10^−1^	2.00 × 10^1^	3.00 × 10^−3^	3.00 × 10^−3^

**Table 4:** Descriptive statistics of eight element concentrations in surface soils (*N* = 60, unit mg/kg).

Elements	Mean	Minimum	Maximum	Median	Variance	Standard Deviation	Coefficient of Variation
Hg	0.187	0.037	1.280	0.145	0.197	0.039	105.348
As	17.560	4.010	31.100	18.600	6.170	38.074	35.137
Cr	77.197	9.040	128.000	89.100	32.375	1048.110	41.938
Cu	56.812	7.000	130.000	61.650	26.276	690.428	46.251
Ni	38.307	6.600	62.800	45.850	15.272	233.249	39.867
Pb	50.671	4.370	225.000	42.550	32.445	1052.683	64.031
Zn	131.333	34.400	444.000	134.000	55.590	3090.223	42.328
Cd	0.436	0.025	0.840	0.470	0.202	0.041	46.330

**Table 5:** Concentrations of eight elements in Zhongshan and other cities of the Guangdong–Hong Kong–Macao Greater Bay Area.

City	Mean Concentrations of Elements (mg/kg)								References
	Hg	As	Cr	Cu	Ni	Pb	Zn	Cd	
Zhongshan	0.2	17.6	77.2	56.9	38.3	50.7	131.3	0.4	This study
Dongguan	0.7	7.1	40.8	48.3	42.8	61.8	92.0	0.1	[32]
Guangzhou	0.7	10.9	64.7	24.0	12.35	58.0	162.6	0.3	[33]
Shenzhen	0.2	16.9	81.6	87.8	88.4	163.5	85.3	0.1	[34]
Zhuhai	-	-	36.8	43.0	26.8	40.4	189.7	0.5	[35]
Foshan	0.4	16.2	80.8	49.5	33.2	52.6	128.1	0.6	[36]
Huizhou	0.2	15.1	52.8	-	-	46.2	-	0.2	[36]
Jiangmen	0.3	12.2	75.6	-	-	46.5	-	0.2	[36]
Zhaoqing	-	35.2	-	33.5	30.5	33.8	54.9	-	[37]
Hong Kong	-	13.3	-	17.1	-	56.9	55.2	1.0	[38]
Macao	-	-	-	313	51	76	385	2.0	[35]
Pearl River Delta Soil background values	0.1	25.0	77.0	32.0	28.0	60.0	97.0	0.1	[25]

**Table 6:** Matrix of correlation coefficient of eight elements in surface soils of northern Zhongshan.

Elements	Hg	As	Cr	Cu	Ni	Pb	Zn	Cd
**Hg**	1							
**As**	0.234	1						
**Cr**	0.171	0.777 **	1					
**Cu**	0.170	0.501 **	0.719 **	1				
**Ni**	0.201	0.726 **	0.937 **	0.769 **	1			
**Pb**	0.008	0.077	0.323 **	0.042	0.184	1		
**Zn**	0.018	0.218	0.357 **	0.698 **	0.533 **	0.474 **	1	
**Cd**	0.134	0.628 **	0.742 **	0.678 **	0.800 **	0.094	0.509 **	1

** correlation was significant when the confidence level (dual-test) was 0.01.

**Table 7:** Eigenvalues and cumulative contribution rates (*N* = 60).

Factors	Initial Eigenvalue			Extract Square Sum Loading			Rotation Square Sum Loading		
	Total Special Value	Percentage of Variance	Cumulative Variance Contribution Rate %	Total Special Value	Percentage of Variance	Cumulative Variance Contribution Rate %	Total Special Value	Percentage of Variance	Cumulative Variance Contribution Rate %
F1	4.295	53.686	53.686	4.295	53.686	53.686	4.213	52.663	52.663
F2	1.545	19.315	73.000	1.545	19.315	73.000	1.572	19.648	72.311
F3	1.020	12.749	85.749	1.020	12.749	85.749	1.075	13.438	85.749

**Table 8:** Pre- and post-rotation factor load matrices.

Elements	F1		F2		F3	
	Before Rotation	After Rotation	Before Rotation	After Rotation	Before Rotation	After Rotation
Hg	−0.197	−0.110	0.222	−0.018	0.924	0.964
As	0.782	0.779	-0.230	−0.078	−0.160	−0.279
Cr	0.925	0.953	−0.280	−0.158	0.070	−0.081
Cu	0.852	0.834	0.263	0.327	0.203	0.182
Ni	0.961	0.962	−0.100	0.033	0.016	−0.094
Pb	−0.089	−0.224	0.879	0.903	−0.304	−0.079
Zn	0.611	0.519	0.716	0.778	−0.010	0.104
Cd	0.871	0.864	−0.004	0.108	0.039	−0.041
